# Synaptotagmin 4 and 5 additively contribute to Arabidopsis immunity to *Pseudomonas syringae* DC3000

**DOI:** 10.1080/15592324.2021.2025323

**Published:** 2022-01-21

**Authors:** Soohong Kim, Keunchun Park, Chian Kwon, Hye Sup Yun

**Affiliations:** a Department of Molecular Biology, Dankook University, Cheonan, Korea; b Department of Biological Sciences, Konkuk University, Seoul, Korea

**Keywords:** SYT4, SYT5, VAMP721/722, immunity, *P. syringae* DC3000

## Abstract

Soluble *N*-ethylmaleimide-sensitive factor attachment protein receptors (SNAREs) are essential for vesicle trafficking in plants. Vesicle-associated membrane protein 721 and 722 (VAMP721/722) are secretory vesicle-localized R-SNAREs, which are involved in a variety of biological processes in plants. Compared to VAMP721/722, a VAMP721/722-interacting plasma membrane (PM)-localized Qa-SNARE is engaged in a rather specific physiological process. This indicates that an in vivo regulator controls an interaction between a Qa-SNARE and VAMP721/722 for a specific cellular activity. We previously reported that synaptotagmin 5 (SYT5) modulates the interaction between SYP132 PM Qa-SNARE and VAMP721/722 for Arabidopsis resistance to *Pseudomonas syringae* DC3000. In this study, we show that defense against *P. syringae* DC3000 is compromised in SYT4-lacking plants, which belongs to the same subclade as SYT5. Further elevation of bacterial growth in *syt4 syt5-2* plants compared to either *syt4* or *syt5-2* single mutant suggests that SYT4 and SYT5 play additive roles in Arabidopsis immunity to *P. syringae* DC3000.

Soluble *N*-ethylmaleimide-sensitive factor attachment protein receptors (SNAREs) are the minimal core factors to drive vesicle fusion events in the endomembrane system of eukaryotic organisms including plants.^1,2^ SNAREs are classified into the glutamine-conserved Q-SNARE that is generally localized to a target membrane, and the arginine-conserved R-SNARE that is largely resident in a vesicle.^1,2^ Vesicle-associated membrane protein 721 and 722 (VAMP721/722) R-SNAREs are localized to mobile intracellular compartments, the plasma membrane (PM), and trans-Golgi network (TGN),^3,4^ which supports their function in vesicle trafficking between PM and TGN. VAMP721/722 were originally identified to form a SNARE complex with PM-localized SYP121 Qa-SNARE and SNAP33 Qbc-SNARE for immunity to powdery mildew fungi as well as growth in Arabidopsis.^3^ However, later works revealed that VAMP721/722 are also important for other biological processes such as cell division, root hair growth, and responses to biotic and abiotic stresses.^5–10^

VAMP721/722 are known to interact with PM-localized SYP111 for cytokinesis,^5^ SYP123 and SYP132 for root hair growth,^6^ and SYP121 and SYP132 for immunity.^3,10,11^ This indicates that a specific biological function of VAMP721/722 is determined by their interaction with a functionally specialized PM Qa-SNARE. Interestingly, VAMP721/722 promiscuously form SNARE complexes with the above-mentioned PM Qa-SNAREs in vitro.^3,6,10^ This additionally indicates that a regulator might control the specific interaction between VAMP721/722 and a PM Qa-SNARE in plants. Indeed, such regulators were found to modulate interactions between VAMP721/722 and PM Qa-SNAREs. KEULE (also called SEC11) controls SNARE complex formation of VAMP721/722 with SYP111 during cytokinesis, but with SYP121 during vegetative growth in Arabidopsis.^12,13^ GNOM ADP-ribosylation factor-guanine nucleotide exchange factor (ARF-GEF) is required for focal accumulation of SYP121 at fungal entry sites in Arabidopsis,^14^ likely for concentrated secretion of immune molecules to fungal attacking area by interacting with VAMP721/722. Synaptotagmin 1 (SYT1) regulates SYP121 abundance in Arabidopsis,^15^ likely to maintain the homeostatic level of SYP121 to interact with VAMP721/722 during immune responses. We recently found that SYT5 is required for Arabidopsis resistance to *Pseudomonas syringae* DC3000^16^. Promoted in vitro interaction between SYP132 and VAMP722 by SYT5 but reduced in planta interaction between SYP132 and VAMP721/722 in *syt5* plants^16^ suggest that SYT5 is at least partly responsible for Arabidopsis resistance to *P. syringae* DC3000 by regulating SYP132-VAMP721/722 interaction.

Among five SYTs in Arabidopsis, SYT4 and SYT5 are grouped into the same subclade (Figure 1).^17^ We previously found an immune function of SYT5 to *P. syringae* DC3000^16^. In the present study, we investigated whether or not SYT4 also has a similar immune activity in Arabidopsis. For this, we isolated a homozygous T-DNA-inserted *syt4* mutant (GABI_215E11) (Figure 1), which was confirmed by no *SYT4* transcript in RT-PCR (Figure 2(a)). To test any redundancy or additivity in Arabidopsis immunity between SYT4 and SYT5, we tried to generate *syt4 syt5* double mutant by crossing *syt4* and *syt5* single mutants. Although SYT5 protein was not detected in two independent *syt5-1* and *syt5-2* plants as previously reported (Figure 2(b)), a slight amount of *SYT5* transcripts was found in *syt5-1* plants (Figure 2(a)). Therefore, we crossed *syt4* and *syt5-2* plants to generate *syt4 syt5-2* double mutant plants (Figures 2(a) and 1(b)). We then dip-inoculated those *syt4, syt5-2*, and *syt4 syt5-2* plants with *P. syringae* DC3000. As we previously reported, we found elevated bacterial growth in *syt5-2* plants compared to WT (Figure 2(c)). We also found more bacterial growth in *syt4* plants than WT, which is comparable to that in *syt5-2* plants (Figure 2(c)). Interestingly, bacterial growth was further elevated in *syt4 syt5-2* plants compared to that in either single mutant (Figure 2(c)). This suggests that SYT4 and SYT5 additively involve in Arabidopsis resistance to *P. syringae* DC3000.

**Figure 1.**
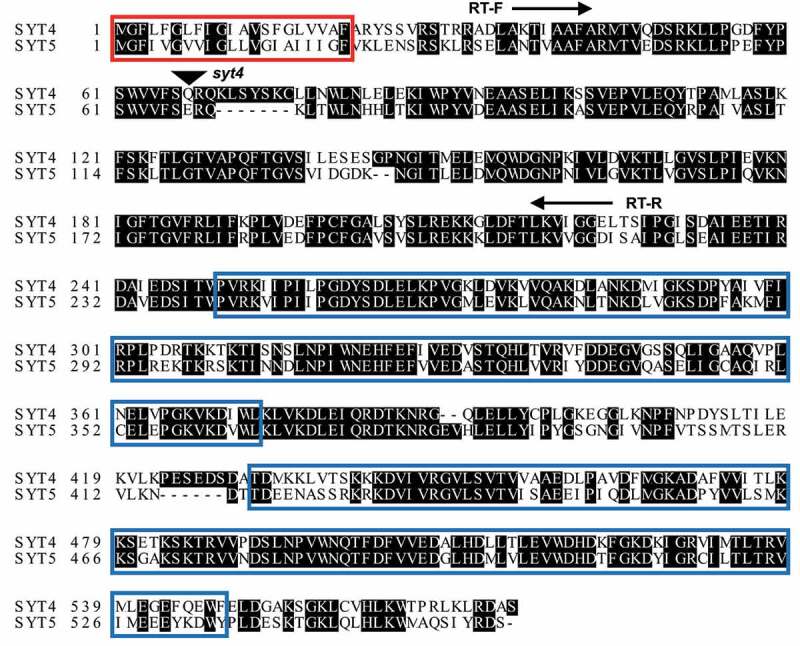
Comparison of SYT4 and SYT5 amino acid sequences.

Amino acid sequences of SYT4 and SYT5 are aligned by ClustalW. The T-DNA insertion site in *syt4* mutant is indicated by a black triangle. Primers used for RT-PCR to detect *SYT4* transcripts are shown as arrows (RT-F, 5ʹ-AGACCATTGCTGCATTTGCAA; RT-R, 5ʹ-TTAATTCACCACCTATAACTT). Red box, transmembrane motif; blue boxes, C2 domains.

**Figure 2.**
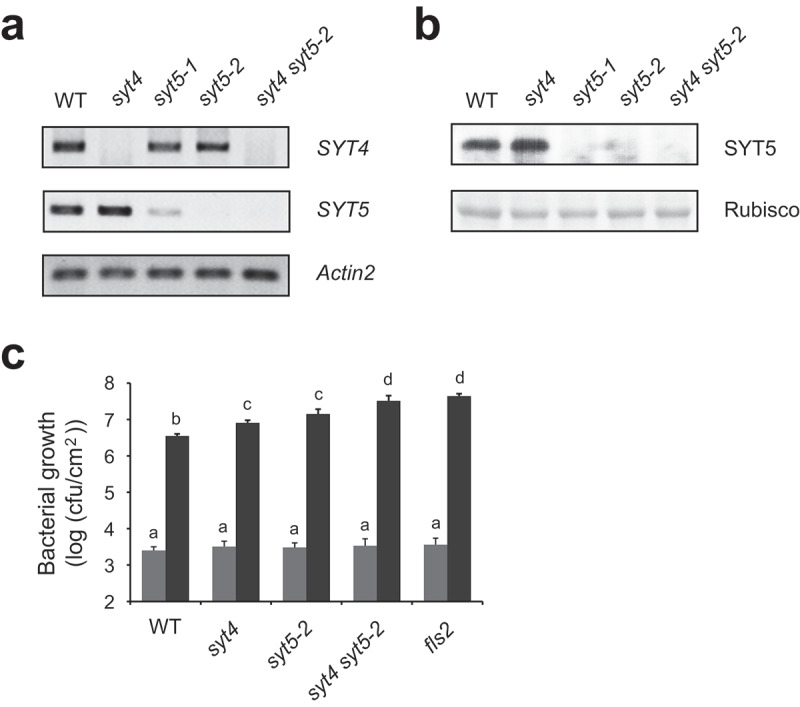
Requirement of both SYT4 and SYT5 for plant resistance to *P. syringae* DC3000.

(a) Transcriptional analysis of *SYT4* and *SYT5* genes in *syt4, syt5-1, syt5-2*, and *syt4 syt5-2* plants. RNA extracts from the indicated genotype plants were subject to RT-PCR using primers to amplify a part of respective *SYT4* or *SYT5* transcripts. *Actin2* was amplified as a control. (b) SYT5 steady-state levels in *syt4, syt5-1, syt5-2*, and *syt4 syt5-2* plants. Protein extracts from the indicated genotype plants were subject to immunoblot with anti-SYT5 antibody. Equal loading was visualized by Rubisco. Note that the used anti-SYT5 antibody can distinguish SYT5 from SYT4. (c) Additive effect of SYT4 and SYT5 on Arabidopsis disease resistance to *P. syringae* DC3000. The indicated genotype plants grown in soil for 4–5 weeks were dip-inoculated with *P. syringae* DC3000 (1 × 10^7^ cfu). Bacterial growth in plant leaves was measured at 3 hours (gray) or 3 days (black) post inoculation. Error bars represent mean ± SE from four biological replicates. Different letters indicate significant differences (P < .01; Student’s *t*-test).

VAMP721/722 are secretory vesicle-directing R-SNAREs to the PM in plants. Their engagement in diverse biological processes indicates that they are major R-SNAREs to drive exocytosis in plants. However, VAMP721/722-interacting PM Qa-SNAREs have rather specific physiological functions such as cell division, growth, and stress responses in plants.^3,5,6,10^ Thus, VAMP721/722 might participate in a specific cellular activity by preferentially interacting with a PM Qa-SNARE. This can be achieved by a regulator that can control the interaction between VAMP721/722 and PM Qa-SNAREs. We recently reported that SYT5 is required for Arabidopsis immune responses to *P. syringae* DC3000 by controlling the interaction between VAMP721/722 and the PM-localized SYP132 Qa-SNARE that is responsible for defense against bacteria.^16^ We here show that SYT4, which belongs to the same subclade with SYT5, is also important for Arabidopsis defense against *P. syringae* DC3000. Interestingly, deletion of SYT4 and SYT5 additively affects Arabidopsis immunity to *P. syringae* DC3000 (Figure 2(c)). Proteomic analysis revealed that VAMP721 and VAMP722 secrete distinct cargos.^18^ In consistence with this, we previously found that VAMP721 and VAMP722 are additively required for Arabidopsis resistance to surface-inoculated *P. syringae* DC3000^16^. Bacterial growth in *syt4 syt5-2* plants is comparable to that in *fls2* plants (Figure 2(c)). Therefore, it is likely that SYT4 and SYT5 differentially regulate the interaction between SYP132 and VAMP721/722 during immune responses to *P. syringae* DC3000. With this, plants can discharge entire immune molecules, parts of which are transported differently by VAMP721 and VAMP722 vesicles, for full resistance to *P. syringae* DC3000. The distinct and additive immune activity of SYT4 and SYT5 might be attributed to the variant region between two C2 domains between SYT4 and SYT5 (Figure 1). It is also of interest to test whether the targeting of FLS2 to the PM is modified in SYT4/SYT5-deficient plants.
